# Characterization of severity of hemolytic disease of the fetus and newborn due to Rhesus antigen alloimmunization

**DOI:** 10.1016/j.xagr.2024.100439

**Published:** 2025-01-09

**Authors:** Alexis A. Krumme, Robert Y. Suruki, Clair Blacketer, Jill Hardin, Joel N. Swerdel, May Lee Tjoa, Kara B. Markham

**Affiliations:** 1 Global Epidemiology, Janssen Research & Development, Titusville, NJ (Krumme, Suruki, Blacketer, Hardin, and Swerdel); 2 Johnson & Johnson, Cambridge, MA (Tjoa); 3 College of Medicine, University of Cincinnati, Cincinnati, OH (Markham).

**Keywords:** alloantibodies, alloimmunization, epidemiology, hemolytic disease of the fetus and newborn, severity, Rhesus D antigen, Rhesus

## Abstract

**BACKGROUND:**

Clinical manifestations of hemolytic disease of the fetus and newborn include anemia, hyperbilirubinemia, hydrops fetalis, kernicterus, and fetal or neonatal demise. More than 50 antibodies are linked to hemolytic disease of the fetus and newborn, with Rhesus (including D and c) and Kell antigens carrying the highest risk of disease. To date, a multicenter, comprehensive evaluation of hemolytic disease of the fetus and newborn due to Rhesus antigen alloantibodies in the United States has not been undertaken.

**OBJECTIVE:**

This study aimed to implement a novel severity index to characterize the real-world disease spectrum of hemolytic disease of the fetus and newborn due to alloantibodies from the Rhesus class.

**STUDY DESIGN:**

This retrospective cohort study was conducted in neonates with commercial insurance available through Optum's deidentified Clinformatics® Data Mart Database (Clinformatics®) and Merative MarketScan® Commercial Claims and Encounters (CCAE) Database from 2000 to 2022. Neonatal and maternal records were linked algorithmically by shared family identifier. A hierarchical severity index was developed for neonates with a Rhesus antigen hemolytic disease of the fetus and newborn diagnosis code in the first 30 days of life, using antenatal and neonatal diagnoses and treatments: *Severe* (neonatal death, hydrops fetalis, intrauterine transfusion); *Moderate* (neonatal exchange transfusion); *Mild* (neonatal simple transfusion); *Minimal* (neonatal phototherapy or hyperbilirubinemia). Maternal, antenatal, and perinatal demographic and clinical characteristics were summarized descriptively by severity.

**RESULTS:**

In Clinformatics® and Commercial Claims and Encounters Database, respectively, 1927 and 1268 neonates met the inclusion criteria. Most (93.1% Clinformatics®; 93.5% CCAE Database) displayed minimal severity, although in both databases nearly 40% of these neonates still required phototherapy. More neonates were mildly affected (3.3% Clinformatics®; 2.2% Commercial Claims and Encounters Database) than moderately (1.0% Clinformatics®; 1.1% Commercial Claims and Encounters Database). In Clinformatics® and Commercial Claims and Encounters Database, respectively, severe hemolytic disease of the fetus and newborn affected 2.6% and 3.2% of neonates, 54% and 46% of whom received exchange or simple transfusions. Severely affected neonates were more commonly delivered by cesarean delivery and at a lower gestational age (34.1 weeks Clinformatics®; 35.4 weeks Commercial Claims and Encounters Database) than those minimally affected (38.5 weeks Clinformatics®; 38.4 weeks Commercial Claims and Encounters Database).

**CONCLUSION:**

Results across 2 real-world US databases found that although most neonates affected by hemolytic disease of the fetus and newborn due to Rhesus antigen alloantibodies did not require intervention beyond phototherapy, nearly 7% experienced disease severity requiring invasive intervention or resulting in neonatal mortality. More severely affected neonates had lower gestational age at birth, higher rates of cesarean delivery and neonatal intensive care unit admission, and longer length of hospital stay at birth compared with minimally affected neonates. The HDFN Severity Index is a useful tool to characterize hemolytic disease of the fetus and newborn and may be valuable alongside guideline-driven care in subsequent pregnancies.

**AJOG Global Reports at a Glance**

**Why was this study conducted?**

To date, a comprehensive evaluation of severity of hemolytic disease of the fetus and newborn (HDFN) due to Rhesus antigen alloantibodies in the United States has not been undertaken.

**Key findings**

Results across 2 databases of commercially insured pregnant individuals and their newborns indicated that although most affected neonates did not require intervention beyond phototherapy, nearly 7% of cases were fatal or required invasive interventions. Over the last 20 years, the relative proportion of neonates with minimal clinical manifestations has increased, whereas the absolute number of those with more severe disease has remained stable.

**What does this add to what is known?**

This was a large study in the United States that examined the full spectrum of disease severity, confirmed findings from smaller studies, and demonstrated important trends in incidence and management of HDFN over 20 years.

## Introduction

Hemolytic disease of the fetus and newborn (HDFN) is a rare, potentially life-threatening condition of pregnancy, caused by maternal–fetal red blood cell antigen incompatibility.^1^, ^2^, ^3^, ^4^ HDFN has a range of clinical manifestations, including anemia, hyperbilirubinemia, hydrops fetalis, kernicterus, and fetal or neonatal demise. Treatment options for HDFN are limited and invasive, including intrauterine and neonatal blood transfusions. Alloantibodies carrying the highest risk of HDFN are predominantly in the Rhesus antigen class, including D and c, whereas E, e, and C confer a moderate risk.^5^^,^^6^

To date, most studies of HDFN epidemiology in the United States have been from tertiary care referral centers, results of which may not be generalizable to the entire US population.^7^, ^8^, ^9^ One recent study used the US National Inpatient Sample of hospital discharges to estimate nationwide HDFN prevalence and treatments in neonates using International Classification of Diseases (ICD)-9 diagnostic and procedure codes up until 2010.^10^ This study period may not capture modern management of HDFN, which includes the use of noninvasive fetal Doppler ultrasound to monitor and diagnose fetal anemia, introduced around 2006. A recent study of alloimmunized pregnancies from the Intermountain Health system found that the predominance of morbidity from HDFN was caused by Rhesus antigen alloantibodies.^11^

This study aimed to characterize and compare the current spectrum of disease severity of HDFN due to Rhesus antigen alloantibodies—including D, C, c, E, and e—in the United States over a 23-year period since 2000. This study used administrative commercial health insurance claims data from pregnancy individuals and their infants and implemented a novel HDFN severity index to hierarchically assign and compare characteristics by disease severity.

## Materials and methods

### Data sources

This study includes data from 2 databases of commercial health care insurance claims in the United States for the years 2000 to 2022: the deidentified Clinformatics® Data Mart Database (Optum, Eden Prairie, MN) and the MarketScan Commercial Claims and Encounters Database (CCAE) (Merative, Ann Arbor, MI). Both contain patient-level, encounter-based, longitudinal records, and include inpatient and outpatient diagnoses, procedures, and outpatient prescription dispensing records. The databases were transformed to the OMOP (Observational Medical Outcomes Partnership) Common Data Model, which provides a standardized representation of database structure and clinical content.^12^ Use of these databases was reviewed by the New England Institutional Review Board and determined to be exempt from broad institutional review board approval.

### Cohort definition

Because direct measures of gestational age are generally not available in these data, a pregnancy algorithm was implemented to identify pregnancies, their timing, duration, and outcome (live birth, stillbirth, abortions, ectopic pregnancy) using markers from antenatal care.^13^ Although more severe HDFN diagnosed antenatally could be recognized through intrauterine transfusion (IUT) procedure codes or hydrops fetalis diagnoses, milder cases or cases not recognized until after birth are imperfectly captured in these data using antenatal HDFN diagnosis codes. As a result, we required a diagnosis of HDFN due to Rhesus antigen alloantibodies on the infant's record (773.0 for ICD-9; P55.0 for ICD-10) within 30 days of birth. However, because not all pregnancies can be algorithmically linked to infant records through a shared family identifier,^14^ pregnancies ending in nonlive births would be overrepresented relative to those ending in live birth and linked to infant records. Thus, to appropriately capture the spectrum of HDFN disease severity, our study was limited to live births. In addition, the cohort was restricted to singleton births with an antenatal record of an obstetrical blood panel at any time during pregnancy. The latter was required to ensure that a minimal level of antenatal care was received and that more complete outpatient laboratory data were available for included patients. Finally, only patients affected by HDFN, as defined by the HDFN Severity Index criteria, were included.

### Outcome

The HDFN Severity Index is a novel, hierarchical index of disease severity: (1) *Severe*: neonatal death, hydrops fetalis, or intrauterine transfusion; (2) *Moderate*: neonatal exchange transfusion and no *Severe* manifestations; (3) *Mild*: neonatal simple transfusion and no *Severe* or *Moderate* manifestations; and (4) *Minimal*: neonatal phototherapy or hyperbilirubinemia and no *Severe, Moderate*, or *Mild* manifestations. Coding algorithms (Supplemental Table S1) were developed through literature search and expert clinician review.

### Statistical analysis

The unit of analysis comprised the mother–infant linked pair of records representing antenatal and postnatal experiences. The distribution of disease severity, as measured by the HDFN Severity Index, was calculated as the proportion of cases in each category both overall and by 5-year calendar intervals. Infants assigned to the highest severity category could have also received treatments associated with other categories. The number of HDFN Severity Index categories for which treatments were received was also estimated. For descriptive antenatal, perinatal, and postnatal characteristics (coding definitions, Supplemental Table S2), means and standard deviations were used for continuous variables, and counts and percentages for categorical variables. Characteristics across severity levels were compared using standardized mean differences.

Trimesters of pregnancy were calculated on the basis of the number of days since the estimated last menstrual period: trimester 1 (0–91 days), trimester 2 (92–182 days or delivery date), and trimester 3 (183 days–delivery date). The perinatal period was defined as the time from the maternal hospital admission for delivery until the infant's hospital discharge. The postnatal period was defined as the time from the infant's discharge date up to 120 days after delivery or disenrollment from the database, whichever occurred first.

## Results

Among 1,864,827 mother–infant linked pairs in Clinformatics®, 6060 (0.3%) had an infant diagnosis code of Rhesus antigen HDFN. Of these, 58% had a record of an obstetrical blood test in the data. In CCAE, among 2,744,253 mother–infant linked pairs, 4881 (0.2%) had an infant Rhesus HDFN diagnosis code. Of these, 47% had an obstetrical blood test in the data. The final cohort with at least 1 severity index criterion was 1927 in Clinformatics® and 1268 in CCAE.

Most neonates experienced *Minimal* severity (93.1% Clinformatics®; 93.5% CCAE), nearly 40% of which required phototherapy (Table 1). More neonates were affected by *Mild* HDFN (3.3% Clinformatics®; 2.2% CCAE) than by *Moderate* HDFN (1.0% Clinformatics®; 1.1% CCAE). In Clinformatics® and CCAE, *Severe* HDFN affected 2.6% and 3.2% of neonates, respectively, 54% and 46% of whom also received exchange or simple transfusions. Among patients with *Severe* HDFN, most or all had at least 1 IUT (82% Clinformatics®; 100% CCAE), whereas fewer than one-quarter had fetal or neonatal hydrops (24% Clinformatics®; 10% CCAE). No neonatal deaths were observed in CCAE, whereas 8% of neonates with *Severe* HDFN in Clinformatics® died.

**Table 1:** HDFN *S*everity Index

Characteristic	Clinformatics	CCAE
HDFN Severity Index, n (% total)	1927 (100)	1268 (100)
Severe	50 (2.6)	41 (3.2)
Moderate	20 (1.0)	14 (1.1)
Mild	63 (3.3)	28 (2.2)
Minimal	1794 (93.1)	1185 (93.5)
Number of severity categories for which patients meet criteria, n (% total)		
1	1807 (93.8)	1207 (95.2)
>1	120 (6.2)	61 (4.8)

*CCAE*, Commercial Claims and Encounters Database; *HDFN*, hemolytic disease of the fetus and newborn.

In Clinformatics® and CCAE, 6.2% and 4.8% of neonates met criteria for >1 HDFN Severity Index category, respectively. Of the affected infants meeting >1 clinical severity index criterion (120 Clinformatics®; 61 CCAE), most had clinical manifestations in combination with phototherapy (68% in Clinformatics®; 59% in CCAE). In Clinformatics® and CCAE, 20% and 30% experienced *Severe* manifestations and received a simple transfusion, respectively, and 10% received both exchange and simple transfusions.

Across 5-year intervals, the relative proportion of neonates experiencing *Minimal* HDFN increased (Figure), whereas the relative proportions of all other severity index categories decreased. Exchange transfusions, alone or in combination with other severity index criteria, were more common in earlier than in later time periods. From 2000 to 2004, 5% of infants in Clinformatics® and 7% in CCAE underwent exchange transfusion, as opposed to 2% and 3%, respectively, from 2005 to 2010, and <1% since 2011 (data not shown).

**Figure:**
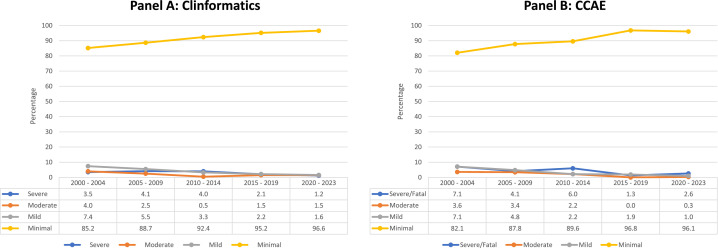
HDFN Severity Index over the study period *CCAE*, Commercial Claims and Encounters Database; *HDFN*, hemolytic disease of the fetus and newborn.

Krumme. Severity of Rhesus antigen Hemolytic Disease of the Fetus and Newborn. Am J Obstet Gynecol Glob Rep 2025.

Antenatal and perinatal characteristics by disease severity are presented in Table 2. Only group B streptococcus testing in the third trimester was more common in the *Minimal* category compared with other severity index categories. Increasing numbers of outpatient visits throughout pregnancy and inpatient visits in trimesters 1 and 2 were recorded with increasing levels of HDFN severity, with the latter reaching 38.0% of pregnancies in Clinformatics® and 34.1% in CCAE for the *Severe* category. In Clinformatics® only, severity categories of *Mild, Moderate*, and *Severe* had a greater proportion of male births (60.0%–65.0%).

**Table 2:** Antenatal and perinatal characteristics by severity of hemolytic disease of the fetus and newborn

Gray cells denote the Clinformatics database, and white cells denote the Commercial Claims and Encounters Database.

*HDFN*, hemolytic disease of the fetus and newborn; *IVIg*, intravenous immunoglobulin; *MCA*, middle cerebral artery; *NICU*, neonatal intensive care unit.

^a^ Redacted because of cell size <5; ^b^ Within 120 days of birth; ^c^ Category includes patients with other/unknown region.

Increasing severity categories showed higher frequency of antibody screening and middle cerebral artery (MCA) Doppler ultrasounds in trimester 2 (Table 2; standardized mean differences are shown in Supplemental Table S3). Only 9.4% of neonates in the *Minimal* category in Clinformatics® and 6.2% in CCAE had both antibody screening and an alloimmunization diagnosis code in pregnancy, which was significantly lower than observed in the *Severe* category (70.0% Clinformatics®; 65.8% CCAE). In the *Minimal* category, only 2.5% in Clinformatics® and 4.0% in CCAE had MCA Doppler performed, as opposed to 62.0% and 82.9%, respectively, in the *Severe* category.

The incidence of different clinical outcomes, including cesarean delivery, gestational age at birth, and mean length of stay, increased with HDFN severity (Table 2; standardized mean differences are shown in Supplemental Table S3). Differences were most pronounced between *Minimal* (cesarean delivery: 32.1% Clinformatics®, 31.4% CCAE; mean gestational age at birth: 38.5 weeks Clinformatics®, 38.4 weeks CCAE; mean length of stay at birth: 3 days Clinformatics® and CCAE) and *Severe* categories (cesarean delivery: 68.0% Clinformatics®, 63.4% CCAE; mean gestational age at birth: 34.1 weeks Clinformatics®, 35.4 weeks CCAE; mean length of stay at birth: 25.1 days Clinformatics®, 12.2 days CCAE). Admission to the neonatal intensive care unit (NICU) occurred for 12.7% and 16.0% of neonates in the *Minimal* category, as opposed to 64.0% and 70.7% in the *Severe* category, in Clinformatics® and CCAE, respectively, including all patients diagnosed with fetal or neonatal hydrops.

## Comment

### Principal findings

Results across 2 databases of commercially insured individuals in the United States indicated that although most neonates affected by HDFN due to Rhesus alloantibodies did not require intervention beyond phototherapy, nearly 7% met criteria for severity, ranging from simple neonatal transfusion to fatal outcomes. Over the last 20 years, the relative proportion of neonates who experienced *Minimal* clinical manifestations has increased; however, absolute numbers of those with more severe disease have been stable. More severely affected neonates had lower gestational age at birth, higher rates of cesarean delivery and NICU admission, and longer length of hospital stay at birth compared with minimally affected neonates.

### Results in the context of what is known

This was a large US study that examined a broad spectrum of severity of HDFN due to Rhesus antigen alloantibodies. There are several challenges to comparing disease severity in HDFN. Notably, these include selected patient populations from specialized referral centers, nonhierarchical definitions of severity that estimate the prevalence of individual interventions, and the absence of more minimal manifestations such as hyperbilirubinemia.^8^^,^^9^ Two US-based studies in more general patient populations have estimated HDFN severity. One study using the National Inpatient Sample estimated that 22% of infants with an HDFN diagnosis code underwent phototherapy, whereas <2% underwent neonatal transfusion.^10^ In a study of antigen-positive neonates receiving care through Intermountain Health, 56% received phototherapy, 9% simple blood transfusions, 3% exchange transfusions, and 5% fetal transfusions^11^; neonates requiring transfusion were all born to mothers with Rhesus antigen alloantibodies. Although an imperfect comparison with the present study measuring severity as a hierarchical index, these results are consistent in magnitude with the current study, where >93% experienced *Minimal* severity and approximately 40% received phototherapy.

The relative proportion of neonates characterized as having *Minimal* HDFN increased over the 20-year study period, whereas the total number of affected neonates in the 3 other severity categories remained stable. This finding is supported by a recent study of obstetrical panel laboratory data from 9.9 million pregnancies in the United States where the annual rate of Rhesus D antigen alloimmunization increased by 43% between 2011 and 2021.^6^ Meanwhile, the rate of pregnancies with critical titers remained stable, suggesting that the increasing rates of alloimmunization have not resulted in increased incidence of more severe HDFN. Possible explanations include increasing ethnic and socioeconomic diversity leading to variable adoption of Rho(D) immune globulin.

Our study found that increasing disease severity and health care utilization corresponded to increasing health care burden of disease. More than half with *Severe* HDFN and slightly fewer than half with *Moderate* and *Mild* disease were admitted to the NICU, and the average length of stay in the hospital was longer for more severely affected neonates. Some of this burden may be mediated by prematurity given that the average gestational age at delivery was <37 weeks for *Severe, Moderate*, and *Mild* HDFN. This is to be expected given standard management of fetal anemia, where premature delivery may be recommended to avoid invasive antenatal intervention while allowing diagnosis and treatment of anemia in the neonate instead. Prematurity also has the potential for serious longer-term health consequences, including respiratory issues and neurocognitive impairment.^15^^,^^16^ Among those with *Minimal* HDFN, the cesarean delivery rate and average gestational age at delivery were similar to those of the general US population, confirming the relatively low burden of disease for these patients.

In CCAE, results by geographic region suggest that severe disease was more common in the Northeast and West regions, whereas no differences were observed by geographic region in Clinformatics®. This finding may reflect differing commercial insurance enrollment patterns between the 2 national databases.

### Clinical implications

HDFN due to Rhesus antigen alloantibodies is estimated to affect approximately 4000 newborns in the Unites States annually.^10^ Because HDFN is asymptomatic in the pregnant person, adherence to screening guidelines and recommendations is of the utmost importance. All pregnancies should be screened for red blood cell alloimmunization, and practitioners should follow strict protocols to guide management of patients with identified alloantibodies. In the study population, fewer than one-fifth had an alloimmunization diagnosis code recorded during pregnancy. Although rates were higher in more severely affected neonates, these results suggest incomplete workup for pregnant individuals at risk of alloimmunization, incomplete use of alloimmunization diagnostic codes in billing, or both.

The health care burden of more severely affected neonates was significant and highlights the challenging decisions that providers must make in balancing the risks of prematurity due to earlier delivery with those of sustained anemia during pregnancy.

In light of the recent American Academy of Pediatrics guidelines for the management of hyperbilirubinemia, which recommend increased thresholds of total bilirubin for exchange transfusion, the decreasing use of exchange transfusions may be expected to continue in the future.^17^ Our finding of decreasing use of exchange transfusion over the last 20 years should be evaluated in other settings. In addition, the increased diagnosis of *Minimal* disease over time is encouraging. Although these neonates did not require significant care, recognition of alloimmunization status and disease risk is important knowledge for a mother and her providers to anticipate and prevent more severe outcomes in future pregnancies.

### Research implications

This study highlights important trends in the severity and burden of HDFN due to Rhesus antigen alloantibodies in the United States over time. It will be important to further characterize emerging trends in HDFN detection and maternal alloimmunization overall, particularly in populations beyond the scope of this study, such as the 41% of pregnancies in the United States insured by state Medicaid programs.

This study implemented a novel, 4-category severity index to hierarchically assign severity to HDFN-affected pregnancies and neonates. Although other studies have characterized the use of individual treatments and treatment combinations, our study provides a clear overview of the spectrum of HDFN disease. Future research can examine the applicability of such an index to other care settings where management of HDFN may differ. In addition, future research can determine the utility of the severity index as a tool to anticipate risk of recurrence and increasing disease severity in subsequent pregnancies.

### Strengths and limitations

This study has several important strengths. The inclusion of >2000 mother–infant pairs across 2 nationwide databases that capture billed diagnoses, procedures, and treatments makes this study a significant contribution to understanding HDFN in the United States. Validated algorithms were used to both define pregnancy start and end date, and to robustly link maternal and infant records.^13^^,^^18^ The 20-year study period enabled the examination of changing diagnosis and treatment patterns. The study relied on ICD-9 and ICD-10 diagnostic codes to identify HDFN due to Rhesus antigen alloantibodies, which do not have validation studies. However, our requirement of at least 1 clinical symptom of HDFN partially mitigates potential misclassification due to rule-out diagnoses. In addition, calendar time trends do not suggest any concerns with the transition from ICD-9 to ICD-10 coding manuals in 2015.

Although insurance claims data reliably capture billable health care encounters, some data are incompletely captured in claims data, such as inpatient medications, laboratory data, and some procedures. For laboratory-based measurements in particular, we attempted to overcome this data missingness through requirement of an obstetrical blood panel test. However, undercapture may still have occurred, particularly for antibody screen and identification tests. In this study, inpatient intravenous immunoglobulin use and antenatal alloimmunization characteristics may be underreported. In addition, mortality is underreported in the CCAE database, which, unlike Clinformatics®, does not link to external death register information. Finally, cause of death cannot be ascertained in the data, although results from Bahr et al^11^ suggest that approximately half of neonatal deaths recorded among antigen-positive neonates were due to alloimmunization itself or sequelae of prematurity.

Several factors could impact the generalizability of results to other populations. In the United States, half of all births annually are covered by state Medicaid programs, whereas our study population was limited to patients with commercial insurance. A recent study highlights the persistent challenges in care coordination in the United States, which may disproportionately affect Medicaid-insured populations.^19^ The HDFN Severity Index is defined by treatments, whose utilization may depend on access to advanced maternal care. Health care access and practice patterns differ globally, potentially resulting in different disease severity classifications, which do not necessarily indicate differing disease severity. The HDFN Severity Index as implemented in these data does not include nonlive birth outcomes, which can affect up to 4% of Rhesus and Kell alloimmunized pregnancies, nor does it include infants who did not survive long enough to be enrolled in insurance.^20^ Therefore, this study underestimates HDFN severity. This study examines HDFN due to Rhesus antigen alloantibodies, whereas HDFN can also be caused by other and multiple alloantibodies, the latter of which is known to cause greater disease severity.^9^

### Conclusions

Across 2 US databases, although most affected neonates did not require intervention beyond phototherapy, nearly 7% met criteria for severity ranging from mild to fatal HDFN. More severe disease corresponded to worse perinatal characteristics and greater health care utilization. The HDFN Severity Index is a useful tool to describe HDFN and may be valuable alongside guideline-driven care in subsequent pregnancies.

## CRediT authorship contribution statement

**Alexis A. Krumme:** Writing – review & editing, Writing – original draft, Project administration, Methodology, Investigation, Conceptualization. **Robert Y. Suruki:** Writing – review & editing, Conceptualization. **Clair Blacketer:** Writing – review & editing, Formal analysis, Data curation. **Jill Hardin:** Writing – review & editing, Formal analysis, Data curation. **Joel N. Swerdel:** Writing – review & editing, Formal analysis, Data curation. **May Lee Tjoa:** Writing – review & editing, Methodology, Conceptualization. **Kara B. Markham:** Writing – review & editing, Supervision, Methodology, Conceptualization.

## Appendix Supplementary materials

Image, application 1

## Footnotes

Supplementary material associated with this article can be found in the online version at doi:10.1016/j.xagr.2024.100439.
