# Supplementary material for: Characterization of severity of hemolytic disease of the fetus and newborn due to Rhesus antigen alloimmunization
Source: AJOG Glob Rep. 2025 Jan 9;5(1):100439. doi: 10.1016/j.xagr.2024.100439 (PMC11872512; doi:10.1016/j.xagr.2024.100439)
Supplement: Supplementary file 1 [file mmc1.docx]

**SUPPLEMENT**

**Table S1: HDFN severity index coding definitions**

| **Clinical diagnosis or procedure** | **Code type** | **Code** | **Description** |
| --- | --- | --- | --- |
| Hydrops fetalis | ICD9 | 773.3 | Hydrops fetalis due to isoimmunization |
|  | ICD10 | O36.20xx | Maternal care for hydrops fetalis, unspecified trimester |
|  | ICD10 | O36.21xx | Maternal care for hydrops fetalis, first trimester |
|  | ICD10 | O36.22xx | Maternal care for hydrops fetalis, second trimester |
|  | ICD10 | O36.23xx | Maternal care for hydrops fetalis, third trimester |
|  | ICD10 | P56.0 | Hydrops fetalis due to isoimmunization |
|  | ICD10 | P56.90 | Hydrops fetalis due to unspecified hemolytic disease |
|  | ICD10 | P56.99 | Hydrops fetalis due to other hemolytic disease |
| IUT | CPT | 36460 | Transfusion, intrauterine, fetal |
|  | ICD9 | 75.2 | Intrauterine transfusion |
|  | ICD10 | 30273H1 | Transfusion of Nonautologous Whole Blood into Products of Conception, Circulatory, Percutaneous Approach |
|  | ICD10 | 30273J1 | Transfusion of Nonautologous Serum Albumin into Products of Conception, Circulatory, Percutaneous Approach |
|  | ICD10 | 30273K1 | Transfusion of Nonautologous Frozen Plasma into Products of Conception, Circulatory, Percutaneous Approach |
|  | ICD10 | 30273L1 | Transfusion of Nonautologous Fresh Plasma into Products of Conception, Circulatory, Percutaneous Approach |
|  | ICD10 | 30273M1 | Transfusion of Nonautologous Plasma Cryoprecipitate into Products of Conception, Circulatory, Percutaneous Approach |
|  | ICD10 | 30273N1 | Transfusion of Nonautologous Red Blood Cells into Products of Conception, Circulatory, Percutaneous Approach |
|  | ICD10 | 30273P1 | Transfusion of Nonautologous Frozen Red Cells into Products of Conception, Circulatory, Percutaneous Approach |
|  | ICD10 | 30273Q1 | Transfusion of Nonautologous White Cells into Products of Conception, Circulatory, Percutaneous Approach |
|  | ICD10 | 30273R1 | Transfusion of Nonautologous Platelets into Products of Conception, Circulatory, Percutaneous Approach |
|  | ICD10 | 30273S1 | Transfusion of Nonautologous Globulin into Products of Conception, Circulatory, Percutaneous Approach |
|  | ICD10 | 30273T1 | Transfusion of Nonautologous Fibrinogen into Products of Conception, Circulatory, Percutaneous Approach |
|  | ICD10 | 30273V1 | Transfusion of Nonautologous Antihemophilic Factors into Products of Conception, Circulatory, Percutaneous Approach |
|  | ICD10 | 30273W1 | Transfusion of Nonautologous Factor IX into Products of Conception, Circulatory, Percutaneous Approach |
|  | ICD10 | 30277H1 | Transfusion of Nonautologous Whole Blood into Products of Conception, Circulatory, Via Natural or Artificial Opening |
|  | ICD10 | 30277J1 | Transfusion of Nonautologous Serum Albumin into Products of Conception, Circulatory, Via Natural or Artificial Opening |
|  | ICD10 | 30277K1 | Transfusion of Nonautologous Frozen Plasma into Products of Conception, Circulatory, Via Natural or Artificial Opening |
|  | ICD10 | 30277L1 | Transfusion of Nonautologous Fresh Plasma into Products of Conception, Circulatory, Via Natural or Artificial Opening |
|  | ICD10 | 30277M1 | Transfusion of Nonautologous Plasma Cryoprecipitate into Products of Conception, Circulatory, Via Natural or Artificial Opening |
|  | ICD10 | 30277N1 | Transfusion of Nonautologous Red Blood Cells into Products of Conception, Circulatory, Via Natural or Artificial Opening |
|  | ICD10 | 30277P1 | Transfusion of Nonautologous Frozen Red Cells into Products of Conception, Circulatory, Via Natural or Artificial Opening |
|  | ICD10 | 30277Q1 | Transfusion of Nonautologous White Cells into Products of Conception, Circulatory, Via Natural or Artificial Opening |
|  | ICD10 | 30277R1 | Transfusion of Nonautologous Platelets into Products of Conception, Circulatory, Via Natural or Artificial Opening |
|  | ICD10 | 30277S1 | Transfusion of Nonautologous Globulin into Products of Conception, Circulatory, Via Natural or Artificial Opening |
|  | ICD10 | 30277T1 | Transfusion of Nonautologous Fibrinogen into Products of Conception, Circulatory, Via Natural or Artificial Opening |
|  | ICD10 | 30277V1 | Transfusion of Nonautologous Antihemophilic Factors into Products of Conception, Circulatory, Via Natural or Artificial Opening |
|  | ICD10 | 30277W1 | Transfusion of Nonautologous Factor IX into Products of Conception, Circulatory, Via Natural or Artificial Opening |
| Neonatal exchange transfusion | CPT | 36450 | Exchange transfusion, blood; newborn |
|  | CPT | 36456 | Partial exchange transfusion, blood, plasma or crystalloid necessitating the skill of a physician or other qualified health care professional, newborn |
|  | ICD9 | 99.01 | Exchange transfusion |
|  | ICD10 | 30243H1 | Transfusion of Nonautologous Whole Blood into Central Vein, Percutaneous Approach |
|  | ICD10 | 30233H1 | Transfusion of Nonautologous Whole Blood into Peripheral Vein, Percutaneous Approach |
|  | HCPCS | S3906 | Transfusion, direct, blood |
| Neonatal simple transfusion | CPT | 36440 | Push transfusion, blood, 2 years or younger |
|  | CPT | 36430 | Transfusion, blood or blood components |
|  | HCPCS | G0460 | Autologous platelet rich plasma for chronic wounds/ulcers, including phlebotomy, centrifugation, and all other preparatory procedures, administration and dressings, per treatment |
|  | HCPCS | P9011 | Blood, split unit |
|  | HCPCS | P9010 | Blood (whole), for transfusion, per unit |
|  | HCPCS | P9039 | Red blood cells, deglycerolized, each unit |
|  | HCPCS | P9021 | Red blood cells, each unit |
|  | HCPCS | P9038 | Red blood cells, irradiated, each unit |
|  | HCPCS | P9016 | Red blood cells, leukocytes reduced, each unit |
|  | HCPCS | P9040 | Red blood cells, leukocytes reduced, irradiated, each unit |
|  | HCPCS | P9022 | Red blood cells, washed, each unit |
|  | HCPCS | C1016 | Blood, leukoreduced, frozen/deglycerol/washed, each unit |
|  | HCPCS | C1018 | Whole blood, leukoreduced, irradiated, each unit |
|  | HCPCS | C1010 | Whole blood or red blood cells, leukoreduced, cmv negative, each unit |
|  | HCPCS | S3906 | Transfusion, direct, blood |
|  | ICD9 | 99.00 | Perioperative autologous transfusion of whole blood or blood components |
|  | ICD9 | 99.03 | Other transfusion of whole blood |
|  | ICD9 | 99.02 | Transfusion of previously collected autologous blood |
|  | ICD10 | 30243P0 | Transfusion of Autologous Frozen Red Cells into Central Vein, Percutaneous Approach |
|  | ICD10 | 30233P0 | Transfusion of Autologous Frozen Red Cells into Peripheral Vein, Percutaneous Approach |
|  | ICD10 | 30243N0 | Transfusion of Autologous Red Blood Cells into Central Vein, Percutaneous Approach |
|  | ICD10 | 30233N0 | Transfusion of Autologous Red Blood Cells into Peripheral Vein, Percutaneous Approach |
|  | ICD10 | 30243H0 | Transfusion of Autologous Whole Blood into Central Vein, Percutaneous Approach |
|  | ICD10 | 30233H0 | Transfusion of Autologous Whole Blood into Peripheral Vein, Percutaneous Approach |
|  | ICD10 | 30243P1 | Transfusion of Nonautologous Frozen Red Cells into Central Vein, Percutaneous Approach |
|  | ICD10 | 30233P1 | Transfusion of Nonautologous Frozen Red Cells into Peripheral Vein, Percutaneous Approach |
|  | ICD10 | 30243N1 | Transfusion of Nonautologous Red Blood Cells into Central Vein, Percutaneous Approach |
|  | ICD10 | 30233N1 | Transfusion of Nonautologous Red Blood Cells into Peripheral Vein, Percutaneous Approach |
| Neonatal phototherapy | ICD9 | 99.83 | Other phototherapy |
|  | ICD9 | 99.82 | Ultraviolet light therapy |
|  | HCPCS | S9098 | Home visit, phototherapy services (e.g., bili-lite), including equipment rental, nursing services, blood draw, supplies, and other services, per diem |
|  | HCPCS | E0202 | Phototherapy (bilirubin) light with photometer |
|  | ICD10 | 6A601ZZ | Phototherapy of Skin, Multiple |
|  | ICD10 | 6A600ZZ | Phototherapy of Skin, Single |
| Neonatal hyperbilirubinemia | ICD9 | 774.1 | Perinatal jaundice from other excessive hemolysis |
|  | ICD9 | 774.6 | Unspecified fetal and neonatal jaundice |
|  | ICD10 | P58.8 | Neonatal jaundice due to other specified excess hemolysis |
|  | ICD10 | P58.9 | Neonatal jaundice due to excessive hemolysis, unspecified |
|  | ICD10 | P59.9 | Neonatal jaundice, unspecified |
|  | ICD10 | R17 | Unspecified jaundice |
|  | ICD10 | R17.0 | Hyperbilirubinemia with mention of jaundice, not elsewhere classified |
|  | ICD10 | R17.9 | Hyperbilirubinemia without mention of jaundice, not elsewhere classified |

**Table S2: Antenatal and Perinatal Characteristics coding definitions**

| **Characteristic** | **Code type** | **Code** |
| --- | --- | --- |
| Anatomical ultrasound | CPT | 76805, 76810, 76811, 76812 |
| Gestational diabetes screening | ICD9 | V77.1 |
|  | ICD10 | Z13.1 |
|  | CPT | 82950, 82951, 82952 |
| Group B Streptoccocus test | ICD9 | V28.6 |
|  | ICD10 | Z36.85 |
|  | CPT | 87653, 87802, 87081 |
| Antibody screening and identification test | CPT | 86850, 86870, 86970, 86880, 86886 |
|  | LOINC | 890-4, 14575-5,15343-7, 34532-3, 34531-4, 1007-4, 1003-3, 1004-1, 1005-8, 1006-6, 1007-4, 1008-2, 50012-4, 50959-6, 51006-5, 51871-2, 52114-6, 54410-6, 55774-4, 55776-9, 56886-5 |
| Maternal alloimmunization | ICD9 | 656.10, 656.11, 656.13, 656.20, 656.21, 656.23, 773.0, 773.1, 773.2, 773.3, 773.4, 773.5 |
|  | ICD10 | O36.01x, O36.09x, O36.19x, O36.11, P55.0, P55.1, P55.8, P55.9 |
| Ultrasound of fetal MCA peak systolic velocity | CPT | 76821 |
| Blood typing, non-ABO antigens | CPT | 86901, 86905, 86906 |
| Cesarean delivery | ICD9 | 74.3, 74.4, 74.99 |
|  | ICD10 | 10D00Z2 |
|  | CPT | 01961, 01968, 01969, 01963, 01962, 59620, 59622, 59615, 58611, 59510, 59618, 59525, 00850, 00857 |

**Table S3: Standardized Mean Differences for Categorically-Defined Antenatal and Perinatal Characteristics**

| **Characteristic** | **Minimal vs. Mild** | **Minimal vs. Moderate** | **Minimal vs. Severe/Fatal** | **Mild vs. Moderate** | **Mild vs. Severe/Fatal** | **Moderate vs. Severe/Fatal** |
| --- | --- | --- | --- | --- | --- | --- |
| **Antenatal care** | | | | | | |
| Anatomical ultrasound, second trimester, n (%) | 0.14 | 0.15 | 0.20 | -0.02 | 0.33 | -0.34 |
|  | -0.03 | -0.30 | 0.03 | 0.27 | -0.06 | 0.32 |
| Gestational diabetes screening, second trimester, n (%) | 0.15 | 0.16 | -0.20 | -0.02 | -0.20 | 0.18 |
|  | -0.04 | 0.19 | 0.34 | -0.23 | -0.24 | 0.01 |
| Group B Streptoccocus test, third trimester, n (%) | 0.67 | 1.00 | 0.80 | -0.28 | -0.12 | -0.16 |
|  | 0.64 | 0.97 | 0.83 | -0.29 | -0.17 | -0.12 |
| Any inpatient stay, first and second trimester, n (%) | -0.16 | -0.18 | -0.87 | 0.02 | 0.71 | -0.69 |
|  | -0.16 | -0.38 | -0.85 | 0.23 | 0.71 | -0.48 |
| Any emergency department visit, first and second trimester, n (%) | 0.30 | 0.12 | -0.05 | 0.18 | 0.35 | -0.17 |
|  | -0.09 | 0.24 | -0.25 | -0.33 | 0.16 | -0.49 |
| **Antenatal alloimmunization and HDFN diagnostic** | | | | | | |
| Antibody screening and identification test, first trimester, n (%) | -0.05 | -0.33 | -0.36 | 0.28 | 0.31 | -0.02 |
|  | -0.53 | -0.77 | -0.94 | 0.22 | 0.36 | -0.14 |
| Antibody screening and identification test, second trimester, n (%) | -0.70 | -1.25 | -0.89 | 0.45 | 0.16 | 0.28 |
|  | -0.45 | -1.33 | -1.48 | 0.77 | 0.88 | -0.09 |
| Maternal alloimmunization, first or second trimester, n (%) | -0.83 | -1.62 | -1.94 | 0.59 | 0.77 | -0.17 |
|  | -0.63 | -1.88 | -2.11 | 0.96 | 1.10 | -0.11 |
| Maternal alloimmunization (first or second trimester) AND antibody screening and identification test (first trimester), n (%) | -0.61 | -1.12 | -1.58 | 0.45 | 0.79 | -0.31 |
|  | -0.70 | -1.53 | -1.59 | 0.68 | 0.72 | -0.03 |
| Ultrasound of fetal MCA peak systolic velocity, second trimester, n (%) | -0.77 | -0.58 | -1.65 | -0.20 | 0.71 | -0.94 |
|  | -0.71 | -1.21 | -2.63 | 0.45 | 1.31 | -0.74 |
| Blood typing, non-ABO antigens, first or second trimester, n (%) | -0.17 | -0.06 | -0.75 | -0.11 | 0.56 | -0.68 |
|  | -0.18 | -0.48 | -0.83 | 0.30 | 0.63 | -0.32 |
| **Perinatal** | | | | | | |
| Cesarean delivery, n (%) | -0.49 | -0.47 | -0.77 | -0.01 | 0.26 | -0.27 |
|  | -0.70 | -0.38 | -0.68 | -0.29 | -0.02 | -0.27 |
| NICU admission | -0.79 | -0.65 | -1.08 | -0.12 | -0.24 | -0.37 |
|  | -0.69 | -1.13 | -1.32 | 0.37 | -0.51 | -0.14 |
| Infant IVIg^1^, n (%) | -0.33 | -0.28 | -0.38 | -0.06 | -0.06 | -0.12 |
|  | -0.42 | -0.52 | -0.39 | 0.11 | 0.03 | 0.14 |
| **Maternal and infant demographic** | | | | | | |
| Infant male sex, n (%) | -0.18 | -0.28 | -0.18 | 0.10 | 0.01 | 0.10 |
|  | -0.01 | -0.30 | 0.07 | 0.29 | 0.07 | 0.37 |
| Infant US Census region, n (%) |  |  |  |  |  |  |
| Northeast | -0.07 | -0.19 | -0.22 | 0.12 | -0.14 | -0.03 |
|  | 0.09 | -0.14 | 0.00 | 0.23 | -0.09 | 0.14 |
| Midwest | 0.18 | 0.16 | -0.25 | 0.02 | -0.43 | -0.41 |
|  | -0.05 | -0.05 | 0.13 | 0.00 | 0.18 | -0.60 |
| South | -0.17 | -0.10 | 0.42 | -0.08 | 0.60 | 0.52 |
|  | -0.04 | -0.18 | 0.24 | 0.14 | 0.27 | 0.42 |
| West | 0.11 | 0.13 | -0.05 | -0.02 | -0.16 | -0.18 |
|  | 0.22 | 0.22 | -0.39 | 0.00 | -0.60 | 0.22 |

^1^ Within 120 days of birth. **Note:** Grey cells denote Clinformatics database; white cells denote CCAE database.
